# Patient-needs-enhanced emergency nursing assessment framework accelerates time-critical care for non-traumatic chest pain

**DOI:** 10.3389/fcvm.2025.1663769

**Published:** 2025-11-25

**Authors:** Yan Sun, Xiaoxia Liu, Lili Shen, Fangfang Zhao, Kaiyun Xu

**Affiliations:** 1 Department of Emergency Medicine, Third Affiliated Hospital of Naval Medical University, Shanghai, China; 2 Department of Emergency Medicine, Marine Corps Hospital of the People’s Liberation Army, Chaozhou, Guangdong, China

**Keywords:** chest pain, emergency nursing, quality improvement, patient-centred checklist, electrocardiogram timing

## Abstract

**Background:**

Non-traumatic chest pain requires rapid Emergency Department (ED) triage, yet adherence to ECG ≤10 min and early troponin targets is inconsistent, standard nursing frameworks seldom prompt patient-needs that affect timeliness and documentation. The aim of this study is to determine whether implementing a patient-needs-enhanced Emergency Nursing Assessment Framework (ENAF), compared with usual care, increases the proportion of ED patients with non-traumatic chest pain receiving a 12-lead ECG within 10 min.

**Methods:**

This prospective single-center quasi-experimental before-after study was conducted in the T Third Affiliated Hospital of Naval Medical University from January 2023 to January 2025 and assigned to a control group and ENAF group. The ENAF group comprised (1) eight hours of nurse training, (2) an ENAF electronic template incorporating mandatory pain, anxiety, information-need and social-support items, and (3) a triage “rapid chest-pain kit”. The primary endpoint was completion of a 12-lead ECG within 10 min of triage; secondary endpoints were door-to-troponin time, ≥2-point pain reduction at 30 min, documentation completeness, ED length of stay (LOS) and 30-day major adverse cardiac events (MACE). Multivariable logistic regression adjusted for age, sex, HEART score, arrival mode and peak ED census.

**Results:**

Of 372 screened patients, 340 met eligibility and were analyzed (170 control, 170 ENAF). Timely ECG completion increased from 60.0% to 78.2% (adjusted odds ratio 2.31, 95% CI: 1.47–3.63; *P* < 0.001). Median door-to-troponin time fell from 50 to 39 min (*P* < 0.001); pain-relief success rose from 45.3% to 61.8% (*P* = 0.002). Documentation completeness improved by ten percentage points (*P* < 0.001) and median ED LOS decreased by 0.8 h (*P* = 0.01). Thirty-day MACE was similar between phases (15.3% vs. 12.9%; *P* = 0.49), and no serious adverse events were attributed to the protocol.

**Conclusions:**

Augmenting ENAF with a structured clinical-needs module significantly accelerates ECG acquisition, improves other process metrics and enhances nursing documentation while maintaining patient safety. Adoption of this nurse-led approach could strengthen ED chest-pain pathways in comparable resource-constrained settings, and multicenter validation are warranted to establish generalizability.

## Introduction

Globally, non-traumatic chest pain is a significant cause of emergency department (ED) visits, with a substantial portion of these cases ultimately being diagnosed as acute coronary syndrome (ACS) or benign conditions. In England and Wales, for instance, chest pain accounts for approximately 2.4% of ED visits (1). In China, the EMPACT study highlights the challenge of managing acute chest pain, with a focus on improving diagnostic accuracy and treatment outcomes for suspected ACS cases (2). The proportion of patients with chest pain who are ultimately diagnosed with ACS varies, but a significant number are found to have non-cardiac causes, emphasizing the importance of accurate diagnosis to avoid unnecessary interventions (3).

Guidelines recommend that an electrocardiogram (ECG) should be performed within 10 min of ED arrival for patients with suspected ACS, and troponin levels should be assessed promptly to facilitate early diagnosis and treatment (4). Delays in these critical steps can lead to missed diagnoses of ST-elevation myocardial infarction (STEMI), increased morbidity and mortality, and prolonged ED stays (5). In China, audits of tertiary centers reveal performance gaps, with regional variations in the management of ACS, such as longer onset-to-first medical contact times and differences in the use of percutaneous coronary intervention (PCI) (6). These disparities highlight the need for standardized protocols and quality improvement initiatives to enhance care delivery and outcomes (6).

Emergency nursing practice plays a critical role in patient care, often serving as the first clinical contact for patients in EDs. However, the variability in assessment quality and documentation deficits can contribute to diagnostic errors and medico-legal risks (7, 8). Frameworks like ABCDE and ISBAR are partially adopted to standardize assessments, but their inconsistent use highlights the need for more comprehensive solutions (9). The Emergency Nursing Assessment Framework (ENAF) was developed to address these issues, featuring a five-step structure: Chief Complaint, Primary Survey, Focused Assessment, Secondary Survey, and Reassessment & Communication (10). Evidence suggests that ENAF improves systematic data capture and handoff fidelity, yet it lacks an explicit focus on patient-centered clinical needs such as pain, anxiety, and informational support (10, 11). Addressing patient-centered needs is crucial, as unmet needs are linked to increased anxiety, persistent pain, and decreased patient satisfaction (12). Empirical data indicate delays in analgesia and communication deficits, particularly in chest pain cohorts (13). Local quality control data from a study hospital reveal that only 60% of ECGs are performed within 10 min, and documentation completeness is at 80 ± 10%, with bottlenecks identified in equipment distance, inconsistent reassessment, and lack of standardized prompts for pain and anxiety (14). The HIRAID framework, an evidence-based approach, has been shown to improve the accuracy of clinical documentation and enhance patient experience by providing a structured method for patient assessment and non-technical skills development (8, 11). Despite these advancements, the integration of patient-centered care into emergency nursing frameworks remains a gap that needs addressing to optimize patient outcomes and satisfaction in acute care settings (11, 15).

Nurse-led intervention in combination with a low-cost electronic template with mandatory fields, a readily accessible rapid-assessment kit and a real-time audit-feedback loop, may optimize patient outcome and satisfaction in high-volume emergency departments. The aim of this study is to determine whether implementing ENAF, compared with usual care, increases the proportion of Emergency Department patients with non-traumatic chest pain receiving a 12-lead ECG within 10 min, and to evaluate effects on door-to-troponin time, early pain relief, documentation completeness, emergency department length of stay, and 30-day major adverse cardiac events.

## Methods

### Study design

We performed a prospective, single-center, before-after (quasi-experimental) study that compared usual nursing care (“Control group”) with a patient-needs-enhanced ENAF protocol (“ENAF group”) in adults presenting to the ED with non-traumatic chest pain. Patients were assigned to the Control or ENAF group by calendar time (all eligible visits before protocol go-live comprised the Control phase; all eligible visits after go-live comprised the ENAF phase). Because allocation occurred at the service level and the intervention was visible (electronic ENAF template and pre-positioned rapid chest-pain kit), randomization and allocation concealment were not applicable. No stratification by HEART risk, arrival mode, or triage acuity was used for assignment; these factors were prespecified for covariate adjustment and subgroup analyses only.

### Ethical consideration

This study was conducted at Third Affiliated Hospital of Naval Medical University from January 2023 to January 2025. The study protocol was approved by the Institutional Review Board of Third Affiliated Hospital of Naval Medical University (IRB No. KY-2022-045) and adhered to the Declaration of Helsinki. Because the intervention was a unit-level workflow change with no investigational drug or device and no patient-level randomization, the IRB did not require trial registration.

#### Consent and eligibility

Written informed consent for data use and 30-day follow-up was obtained from all participants prior to inclusion.

#### Privacy and data security

After deterministic linkage of EMR and laboratory feeds, datasets were de-identified with personally identifying fields removed. Data were stored on an encrypted hospital server with access restricted to the study team.

### Participants

Eligibility required age ≥18 years and a chief complaint of non-traumatic chest pain, tightness or discomfort on ED arrival; exclusions were traumatic pain, pre-hospital ECG-confirmed STEMI with direct cath-lab activation, cardiac arrest on arrival, pregnancy, and inability to provide immediate or delayed consent in the absence of a legally authorized representative, all of which were determined by a triage research nurse who prospectively logged screening outcomes. Consecutive ED arrivals meeting eligibility were prospectively screened by a triage research nurse who logged inclusions and exclusions in real time. Assignment to study phase was determined solely by visit date relative to ENAF implementation. No patient-level sequence generation or concealment was performed.

### ENAF protocol

The multifaceted intervention comprised (1) eight hours of nurse education—four hours of pathophysiology and ENAF theory plus four hours of high-fidelity simulation, (2) an EMR-embedded ENAF template with mandatory fields for pain score, GAD-2 anxiety screening, information-need prompt and social-support query, enforced by electronic “hard stops,” (3) a pre-positioned rapid chest-pain kit (ECG cart, aspirin, glyceryl trinitrate spray, pre-labelled troponin tube) within five meters of triage, and (4) weekly audit-and-feedback huddles displaying run charts; baseline care used unstructured nursing notes and lacked both the template and kit.

Training was delivered by the ED nurse-education team with input from cardiology and the ED informatics nurse for the electronic template. After the 8-h course (4 h didactic on ACS/ENAF; 4 h high-fidelity simulation), post-training competency was verified using a brief knowledge test and a skills checklist with return-demonstration covering ECG initiation, pain and GAD-2 documentation, pre-labeling a troponin tube, and use of the rapid chest-pain kit. Passing required a score ≥[80]% on the knowledge test and correct completion of all pre-specified “must-pass” checklist items. Remediation consisted of targeted coaching and a repeat return-demonstration before independent ENAF use. Attendance and competency sign-off were logged in the departmental education record.

### Outcomes

The primary endpoint was completion of a 12-lead ECG within 10 min of triage registration, captured automatically from ECG time stamps; secondary endpoints were door-to-first-troponin time, ≥2-point pain reduction at 30 min, ENAF documentation completeness (modified nine-item D-CATCH score), ED length of stay (LOS) and 30-day major adverse cardiac events (MACE: all-cause death, non-fatal myocardial infarction or unplanned revascularization), with MACE ascertained by telephone follow-up and provincial health-information-system cross-check.

### Sample-Size calculation

Assuming a baseline ECG ≤10 min rate of 60% and targeting a 15-percentage-point absolute increase to 75%, a two-sided *α* = 0.05% and 80% power yielded 152 patients per phase; anticipating a 10% attrition rate we prospectively enrolled 170 patients in each phase (total = 340).

### Data collection and management

Nightly automated scripts extracted demographic data, timestamps and laboratory results from the Neusoft v9.3 EMR and Cobas laboratory information system into a REDCap 13.2 database, while two blinded abstractors audited 20% of charts for documentation completeness with *κ* > 0.80 agreement; datasets were de-identified, stored on an encrypted hospital server and accessed via two-factor authentication with an immutable audit trail.

#### Anonymity and data protection

After deterministic linkage of EMR and laboratory feeds, datasets were de-identified. Personally identifying fields were removed. Data were stored on an encrypted hospital server with two-factor authentication and an immutable audit trail. Access was limited to the study team.

### Statistical analysis

Analyses were executed in Stata 18.0. Categorical variables compared by *χ*^2^ or Fisher's exact tests and continuous variables by Student's *t*-test or Mann–Whitney *U* as appropriate; multivariable logistic regression estimated adjusted odds ratios (aORs) for timely ECG controlling for age, sex, HEART score, arrival mode and peak hourly ED census, while linear/quantile regression, logistic regression and Cox models addressed secondary outcomes, and interaction terms tested predefined subgroups (HEART strata, arrival mode, triage acuity); robustness was assessed via multiple-imputation, per-protocol and interrupted-time-series sensitivity analyses, with two-sided *P* < 0.05 considered significant.

## Results

### Participant flow and baseline characteristics

**Figure 1:**
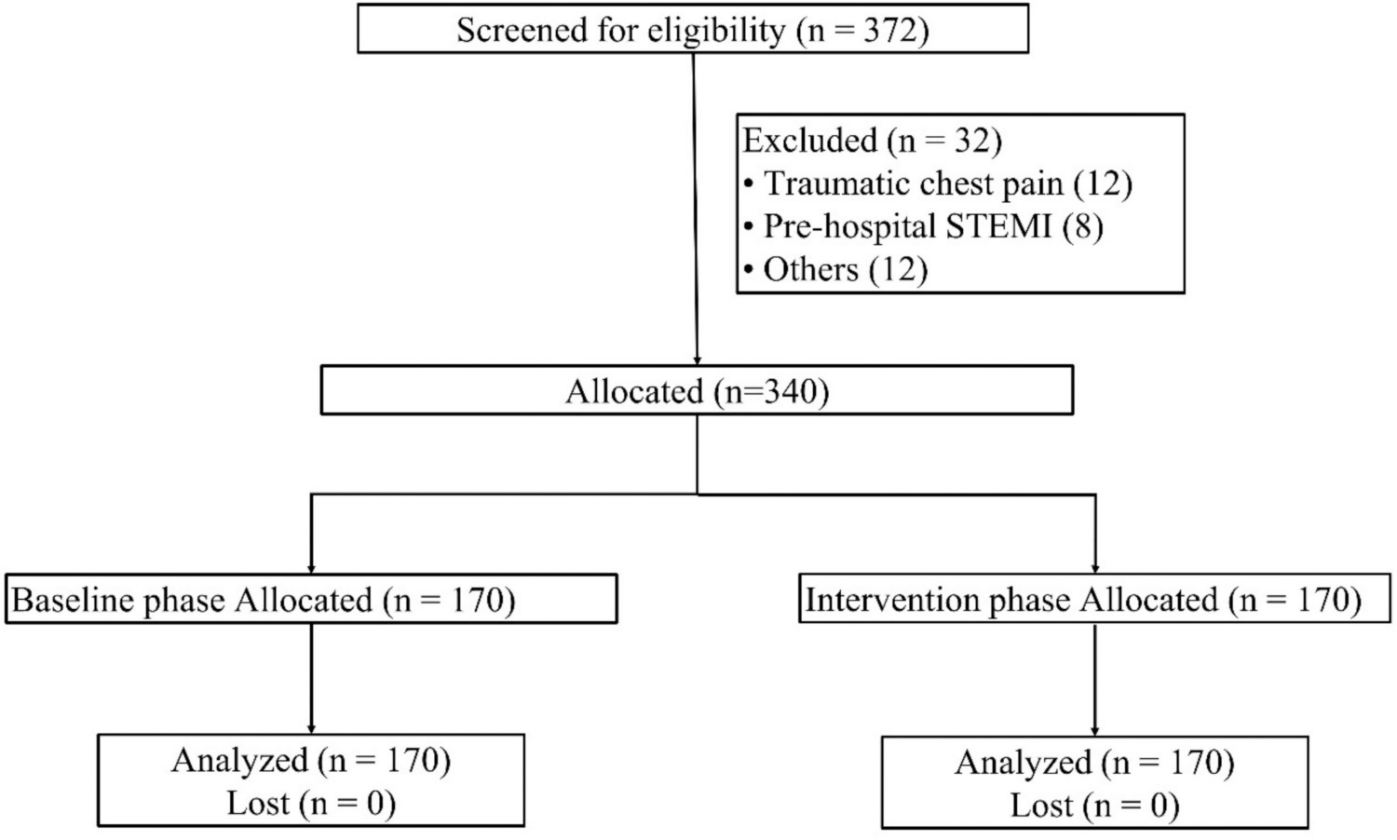
CONSORT-style flow diagram of participant disposition. A total of 372 adult patients with non-traumatic chest pain were screened between January 2023 and January 2025; 32 were excluded (12 traumatic pain, 8 pre-hospital ST-segment-elevation myocardial infarction, 12 other reasons), leaving 340 eligible patients. Allocation was 170 to the baseline phase and 170 to the intervention phase, with zero loss to follow-up or missing primary-outcome data.

From January 2023 to January 2025, 372 adults with non-traumatic chest pain were screened; 32 were excluded (12 traumatic pain, eight pre-hospital STEMI, 12 other), leaving 340 patients—170 in the baseline phase and 170 in the ENAF phase—and none were lost to follow-up (Figure 1). Baseline comparability was excellent (Table 1): mean age 57 ± 14 vs. 56 ± 15 years, 60% male in both cohorts, and no significant differences in ambulance arrivals, HEART ≥4 proportion, initial pain ≥7, or major comorbidities (all *P* > 0.50).

**Table 1:** Baseline characteristics.

Characteristic	Control (*n* = 170)	ENAF (*n* = 170)	*P*-value
Age, mean ± SD (years)	57 ± 14	56 ± 15	0.56
Male, *n* (%)	103 (60.6%)	101 (59.4%)	0.82
Arrival by ambulance, *n* (%)	68 (40.0%)	65 (38.2%)	0.74
HEART ≥ 4, *n* (%)	49 (28.8%)	53 (31.2%)	0.58
Initial pain score ≥7, *n* (%)	87 (51.2%)	84 (49.4%)	0.78
Hypertension, *n* (%)	85 (50.0%)	88 (51.8%)	0.71
Diabetes, *n* (%)	44 (25.9%)	40 (23.5%)	0.64
Prior CAD, *n* (%)	39 (22.9%)	42 (24.7%)	0.68

### Primary outcome—timely ECG acquisition

The ENAF increased the proportion of patients receiving a 12-lead ECG within 10 min from 60.0% (102/170) to 78.2% (133/170), an 18.2-percentage-point absolute gain; unadjusted RR 1.30 (95% CI: 1.10–1.50) and adjusted OR 2.31 (95% CI: 1.47–3.63, *P* < 0.001) (Table 2).

**Table 2:** Primary outcome (timely ECG).

Outcome	Control (*n* = 170)	ENAF (*n* = 170)	Absolute Δ (%)	Relative risk (95% CI)	Adjusted OR^†^ (95% CI)	*P*
ECG ≤10 min, *n* (%)	102 (60.0%)	133 (78.2%)	+18.2	1.30 (1.10– 1.50)	2.31 (1.47– 3.63)	<0.001

† Adjusted for age, sex, HEART score, arrival mode, and peak ED census.

### Secondary outcomes

**Figure 2:**
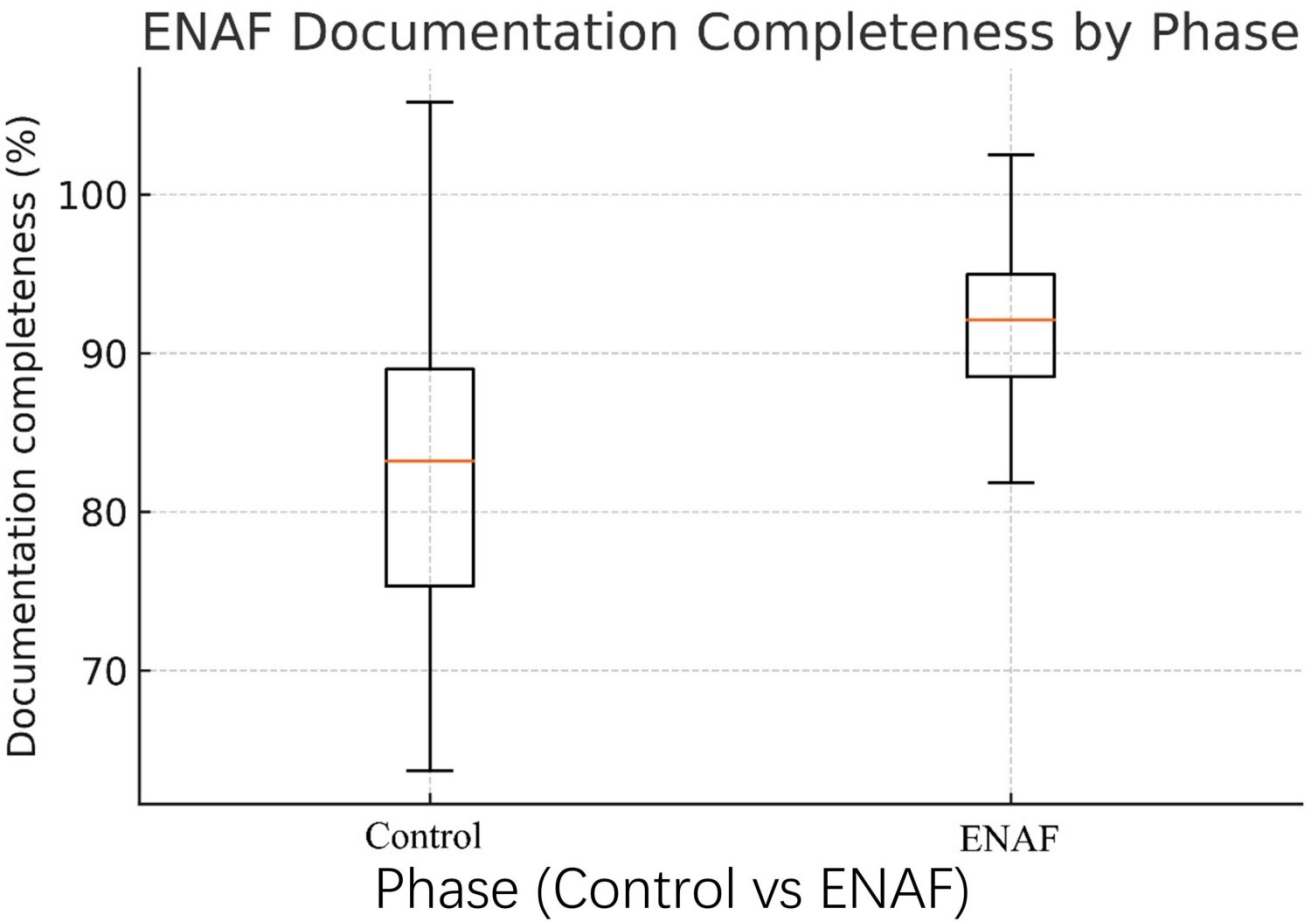
Box-and-whisker plot of ENAF documentation completeness by study phase. Boxes represent the interquartile range (IQR), central lines the median, and whiskers 1.5 × IQR for 170 charts per phase. Median completeness improved from 81% (Control) to 92% (ENAF); outliers are omitted for clarity.

Process metrics improved across the board (Table 3): median door-to-troponin time fell by 11 min (50 → 39 min, *P* < 0.001); pain-relief success at 30 min rose from 45.3% to 61.8% (*P* = 0.002); documentation completeness increased from 82% ± 9% to 92% ± 5% (*P* < 0.001; Figure 2); and median ED length of stay decreased from 5.1 h to 4.3 h (*P* = 0.01), while 30-day MACE was unchanged (15.3% vs. 12.9%, *P* = 0.49).

**Table 3:** Secondary outcomes.

Secondary outcome	Control	ENAF	*P*-value
Door-to-troponin, median (IQR) min	50 (38–65)	39 (30–50)	<0.001
Pain reduction ≥2 at 30 min, *n* (%)	77 (45.3%)	105 (61.8%)	0.002
Documentation completeness, mean ± SD	82 ± 9	92 ± 5	<0.001
ED length of stay (h), median (IQR)	5.1 (3.9–6.4)	4.3 (3.3–5.4)	0.01
30-day MACE, *n* (%)	26 (15.3%)	22 (12.9%)	0.49

### Process fidelity and checklist uptake

The ENAF electronic template was properly closed in 97.4% of ENAF cases and the four-item clinical-needs checklist was fully completed in 95.8%, with template completion time falling from 6.2 min in week 1–4.1 min in week 12 (Supplementary Table S1).

### Subgroup analyses

**Figure 3:**
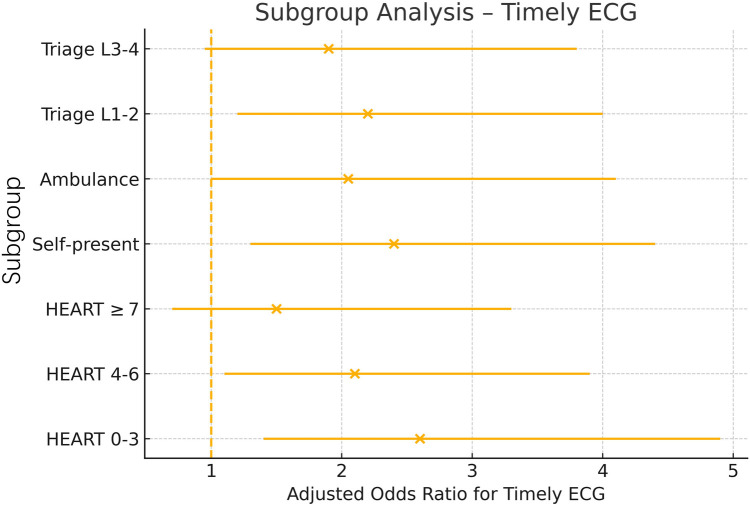
Forest plot of adjusted odds ratios (aORs) for timely ECG across prespecified subgroups. Horizontal lines indicate 95% confidence intervals and solid dots the point aORs comparing intervention vs. baseline within each subgroup (HEART risk strata, arrival mode, triage acuity). The dashed vertical line at aOR = 1.0 denotes no effect; all confidence intervals favor the intervention, and no subgroup shows a significant interaction. HEART, History-ECG-Age-Risk-Troponin score; L, triage level.

Benefits were consistent across predefined strata (Figure 3). Adjusted ORs for timely ECG ranged from 1.50 (HEART ≥7) to 2.60 (HEART 0–3), with no significant interactions by HEART risk, arrival mode, or triage acuity (*P*-interaction >0.10 for all).

### Sensitivity analyses

Multiple imputation for < 2% missing timestamps and a per-protocol analysis excluding eight minor-deviation cases yielded similar results (aOR: 2.24, 95% CI: 1.41–3.55), and an interrupted time-series model demonstrated a level change of +17.6 percentage points immediately after implementation (*P* < 0.001) with no pre-intervention secular trend (Supplementary Table S2).

### Adverse events

No serious adverse events were attributed to the protocol; rates of nitroglycerin-induced hypotension, contrast nephropathy, or medication error were each <1% and comparable between phases (Supplementary Table S3).

## Discussion

This before-after study achieved all prespecified objectives, demonstrating that the patient-needs-enhanced ENAF protocol produced clinically meaningful gains across multiple dimensions of chest-pain care: the proportion of patients receiving an ECG within 10 min rose by 18 percentage points, median door-to-troponin time fell by 11 min, ENAF documentation completeness improved by 10 points, and median emergency-department length of stay shortened by 0.8 h, while 30-day major adverse cardiac events and protocol-related harms remained unchanged. These effect sizes are both statistically robust and operationally significant, indicating that a low-cost, nurse-led, patient-centered workflow can simultaneously accelerate time-critical diagnostics, enhance symptom control and elevate record quality without sacrificing safety.

Our findings both corroborate and extend earlier quality-improvement work on ACS. The 18-percentage-point rise we observed in ECG ≤10 min compares favorably with the 43-percentage-point gain reported when U.S. centers created a dedicated ECG room and hired technicians (16), but it was achieved without extra staff by combining an electronic hard-stop template, a rapid-assessment kit and weekly audit feedback. Pre-hospital ECG programs likewise shorten diagnosis-to-balloon times, yet remain under-utilized even though compelling single-case reports show life-saving impact in ST-segment-elevation myocardial infarction (17). On the biomarker side, we trimmed door-to-troponin by 11 min, echoing rapid rule-out protocols that leverage high-sensitivity cardiac troponin to expedite non-ST-elevation myocardial infarction decisions (18, 19); our approach, however, embeds specimen collection into the same nursing checklist that triggers the ECG, a design choice that may explain why our effect size exceeds those of earlier Chinese chest-pain-center accreditations. Although point-of-care biomarker testing is emerging for ambulance use (20), our data show that simple in-hospital workflow redesign can deliver comparable speed gains while awaiting wider adoption of mobile assays. Importantly, we also improved symptom control and documentation completeness—dimensions often overlooked in process studies—highlighting that a unified checklist can address pain, anxiety and record quality without sacrificing timeliness.

The mechanisms underpinning these gains are consistent with Cognitive Load Theory. By imposing electronic hard stops, the ENAF template removes extraneous memory demands and guides nurses through a fixed sequence of actions, thus preventing omissions at peak workload (21, 22). Visual prompts embedded in the template and the physical co-location of ECG and blood-draw equipment further reduce intrinsic load and accelerate task initiation, mirroring experimental work in STEMI protocols (23). Continuous audit-feedback loops reinforce correct behavior and have shown durable effects in ACS QI projects (24); the sustained upward trend across three ENAF months makes a transient Hawthorne effect unlikely despite the before-after design (22).

Clinically, these results argue for scaling nurse-led ECG programs in high-volume, resource-constrained emergency departments. Cost-effectiveness analyses estimate that structured ECG deployment in urban India costs as little as US $12.65 per quality-adjusted life-year (25), while person-centered care models consistently dominate usual care on both cost and effectiveness over two- to five-year horizons (26, 27). When combined with our low-cost electronic template and audit infrastructure, such programs can be rapidly disseminated—potentially as part of national ED quality indicators or nursing curricula—and do not require physician expansion or capital-intensive technology (28, 29). Crucially, our data dispel the notion that patient-centered assessment slows critical actions; on the contrary, integrating needs appraisal into the first nursing touchpoint expedited every downstream time target and left 30-day major adverse cardiac events unchanged, underscoring both the efficiency and safety of a holistic, checklist-based approach.

The ENAF protocol is practical for high-volume EDs because it relies on existing nursing staff, an 8-h education package, a low-overhead EMR template with hard-stops, and a pre-positioned rapid chest-pain kit near triage. Implementation occurred at the unit level with no additional staff positions. The protocol was associated with an 18.2-percentage-point increase in ECG ≤10 min and a 0.8-h reduction in ED LOS, indicating improved flow without compromise in safety (30-day MACE unchanged). Although not measured formally, high template closure and checklist completion, together with improved pain relief at 30 min, suggest good acceptability. The electronic template sits within triage ENAF, triggers early ECG and pre-labels troponin at first contact, and then hands off to routine clinician decision-making within existing ACS pathways, requiring only minor workflow adjustments.

Although we did not collect cost data, the intervention was intentionally resource-light: a one-day (8-h) nurse training package, an EMR template with hard-stops, a pre-positioned “rapid chest-pain kit,” and brief weekly audit-and-feedback huddles, all delivered with existing staff and infrastructure. These features suggest low marginal costs to implement and maintain. Potential cost offsets, which require formal evaluation, include shorter ED length of stay, fewer workflow delays, and improved documentation quality. Because this study focused on process effectiveness, a future multicenter evaluation should incorporate micro-costing, time-motion, and budget-impact analyses from both hospital and payer perspectives to determine whether the protocol is cost-effective at scale.

This single-center, quasi-experimental design is subject to several limitations. First, the design is vulnerable to secular trends and residual confounding despite prespecified covariate adjustment and sensitivity checks. We cannot exclude unmeasured changes in staffing, case mix, or concurrent initiatives. Second, written informed consent was required. This may have preferentially enrolled patients able to consent, potentially limiting generalizability to populations with communication barriers or severe illness. Third, performance bias is possible because clinical staff could not be blinded to a unit-level workflow change. Fourth, timing measures relied on routine time-stamps. Although missingness was low, small clock drift and documentation variability could affect some secondary outcomes. Fifth, the study was powered for process endpoints. The 30-day MACE analysis was underpowered to detect modest safety differences, and events beyond 30 days were not assessed. Our tertiary urban setting with established informatics support may limit external validity for low-resource emergency departments. We did not collect cost data, our economic comments are therefore conceptual and highlight the need for a formal, prespecified economic evaluation in future multicenter studies. To assess reproducibility across different case mixes, ED volumes, informatics maturity, and staffing models, multicenter studies, ideally cluster-randomized or stepped-wedge designs to manage contamination, are warranted. Such trials should predefine core process and safety outcomes, capture implementation metrics, and embed a concurrent economic evaluation.

Causal confirmation and implementation guidance would benefit from multicenter cluster-randomized or stepped-wedge trials comparing ENAF with standard triage. Key priorities include prespecified subgroup analyses by HEART risk strata, arrival mode, and triage acuity to characterize heterogeneity of effect, a core outcome set for chest-pain triage covering time-to-ECG, door-to-troponin, timely analgesia, documentation quality, ED length of stay, and 90-day safety, implementation-science measures (fidelity, acceptability, time-motion) and cost-effectiveness to inform scale-up in resource-constrained settings, and development of a standardized ENAF training and competency package to enhance reproducibility across platforms and staffing models.

## Conclusions

Embedding a concise, patient-centered clinical-needs module within the Emergency Nursing Assessment Framework markedly accelerated time-critical diagnostics for non-traumatic chest pain and elevated documentation quality without increasing adverse events, demonstrating that comprehensive assessment and rapid care are not mutually exclusive. Given its low marginal resource requirements, multicenter evaluation with an embedded economic analysis is a logical next step to establish generalizability and value.

## Data Availability

The raw data supporting the conclusions of this article will be made available by the authors, without undue reservation.
