# Supplementary material for: Patient-needs-enhanced emergency nursing assessment framework accelerates time-critical care for non-traumatic chest pain
Source: Front Cardiovasc Med. 2025 Nov 25;12:1663769. doi: 10.3389/fcvm.2025.1663769 (PMC12685830; doi:10.3389/fcvm.2025.1663769)
Supplement: Supplementary file 1 [file Table1.docx]

**Supplementary Table S1. Process Fidelity and Checklist Uptake**

| **Metric** | **Week 1** | **Week 4** | **Week 8** | **Week 12** | **Overall*** |
| --- | --- | --- | --- | --- | --- |
| ENAF template closure (%) | 96.0 | 97.0 | 98.2 | 98.5 | 97.4 |
| Clinical‑needs checklist completion (%) | 94.5 | 95.2 | 96.3 | 97.0 | 95.8 |
| Average template completion time (min) | 6.2 | 5.1 | 4.5 | 4.1 | 5.0 |

*Overall = mean of the four time points.
