# Supplementary material for: Patient-needs-enhanced emergency nursing assessment framework accelerates time-critical care for non-traumatic chest pain
Source: Front Cardiovasc Med. 2025 Nov 25;12:1663769. doi: 10.3389/fcvm.2025.1663769 (PMC12685830; doi:10.3389/fcvm.2025.1663769)
Supplement: Supplementary file 2 [file Table2.docx]

**Supplementary Table S2. Sensitivity Analyses for Timely ECG**

| **Analysis** | **Adjusted OR (95% CI)** | **P‑value** |
| --- | --- | --- |
| Main (intention‑to‑treat) | 2.31 (1.47 – 3.63) | < 0.001 |
| Multiple imputation | 2.28 (1.46 – 3.56) | < 0.001 |
| Per‑protocol | 2.24 (1.41 – 3.55) | < 0.001 |
| Interrupted time‑series, level change | +17.6 percentage points | < 0.001 |
