# Supplementary material for: Patient-needs-enhanced emergency nursing assessment framework accelerates time-critical care for non-traumatic chest pain
Source: Front Cardiovasc Med. 2025 Nov 25;12:1663769. doi: 10.3389/fcvm.2025.1663769 (PMC12685830; doi:10.3389/fcvm.2025.1663769)
Supplement: Supplementary file 3 [file Table3.docx]

**Supplementary Table S3. Adverse Events Within 30 Days**

| **Event** | **Baseline**  **(n = 170)** | **Intervention**  **(n = 170)** | **P‑value** |
| --- | --- | --- | --- |
| Nitroglycerin‑induced hypotension | 1 | 1 | 1.00 |
| Contrast nephropathy | 0 | 1 | 0.50 |
| Medication error | 1 | 0 | 0.50 |
| Any serious adverse event (SAE) | 1 | 1 | 1.00 |
